# Role of Serum Lithium Concentrations in Predicting Hyperparathyroidism and Hypercalcemia

**DOI:** 10.1155/ije/6497151

**Published:** 2025-07-24

**Authors:** Noriyoshi Takano, Satoshi Morimoto, Hiroyuki Muraoka, Ken Inada, Katsuji Nishimura, Atsuhiro Ichihara

**Affiliations:** ^1^Division of Hormonal Medicine and Bioregulatory Science, Department of Medicine, Tokyo Women's Medical University, Shinjuku, Tokyo, Japan; ^2^Department of Medicine, Adachi Medical Center, Tokyo Women's Medical University, Adachi, Tokyo, Japan; ^3^Department of Psychiatry, School of Medicine, Kitasato University, Sagamihara, Kanagawa, Japan; ^4^Department of Psychiatry, Tokyo Women's Medical University, Shinjuku, Tokyo, Japan

**Keywords:** bipolar disorder, calcium, lithium, mood disorder, parathyroid, toxicity

## Abstract

**Purpose:** Lithium (Li), which is extensively used in the treatment of mood disorders such as bipolar disorder, has been associated with hyperparathyroidism. However, the relationship between the serum Li concentration and hyperparathyroidism remains unclear. This study aimed (1) to investigate the incidence of hyperparathyroidism and hypercalcemia in consecutive patients treated with Li, (2) to assess the correlation between serum Li concentration and hyperparathyroidism/hypercalcemia, and (3) to establish cutoff values for predicting hyperparathyroidism and hypercalcemia based on serum Li concentration.

**Methods:** This retrospective cross-sectional study was conducted at the Department of Psychiatry and Department of Medicine at Tokyo Women's Medical University. Ninety-seven consecutive individuals without renal impairment and with an estimated glomerular filtration rate (eGFR) equal to or greater than 45 mL/min/1.73 m^2^ were included.

**Results:** Hyperparathyroidism and hypercalcemia were observed in 35.1% and 9.3% of the patients on Li, respectively. The serum Li concentration showed a significant positive correlation with hyperparathyroidism and hypercalcemia, independent of other factors. The cutoff values for predicting hyperparathyroidism and hypercalcemia were 0.52 and 0.62 mEq/L, respectively.

**Conclusions:** This study confirmed that the high incidence of hyperparathyroidism and hypercalcemia in patients treated with Li. Clinicians should be aware that Li treatment may induce hyperparathyroidism, and a serum Li concentration exceeding 0.52 mEq/L may pose an increased risk. Monitoring serum calcium and Li concentrations is recommended in patients undergoing Li treatment.

## 1. Introduction

Lithium (Li) has been extensively used to treat various mood disorders for over 50 years. Li treatment is the first-line maintenance therapy for bipolar disorder (BD) and exhibits remarkable efficacy in preventing and treating manic, depressive, and mixed episodes [1]. Moreover, the treatment is effective in averting depression and mania and contributes to reducing suicide rates among individuals with BD. Li also holds significance as an adjunct to the treatment of major depression, which remains unresponsive to other therapeutic interventions [1, 2]. Li carbonate is also utilized in the management of treatment-resistant schizophrenia. This is primarily because Li is well known as a mood stabilizer and is considered beneficial for alleviating affective symptoms frequently associated with schizophrenia, such as mood disturbances, anxiety, and agitation. Moreover, Li has been suggested to potentiate the therapeutic effects of antipsychotic agents and is, therefore, employed as an adjunctive treatment when antipsychotics alone are insufficiently effective. In addition, several studies have indicated that Li may contribute to a reduction in suicide risk among patients with schizophrenia, and as such, it is sometimes prescribed at the discretion of psychiatrists for individuals deemed to be at elevated risk of suicide. However, Li induces systemic, renal, gastrointestinal, cardiac, and musculoskeletal side effects and triggers endocrine side effects such as hyperthyroidism, hypothyroidism, and hyperparathyroidism [3, 4].

The reported prevalence of hyperparathyroidism in patients taking Li varies significantly, ranging between 2.7% and 50% [5–7]. Similarly, the prevalence of hypercalcemia in subjects receiving Li varies across studies, with reported rates of 3.6% [6] and 7.2% [8]. Measurement of ionized calcium (Ca) has revealed an increased incidence, ranging from 25% to 42.3% [5, 9, 10]. Investigating the prevalence of hyperparathyroidism and hypercalcemia is challenging, likely due to potentially undiagnosed cases, especially in asymptomatic individuals [11].

Hypercalcemia due to Li treatment does not appear to correlate with the duration of therapy, as cases have been reported in patients treated for only 4 weeks [12]. Furthermore, these effects were not associated with the dose or its toxicity [13]. In addition, whether the serum Li concentration is associated with hyperparathyroidism and hypercalcemia remains unclear. Therefore, the present study was conducted with the following objectives: (1) to investigate the incidence of hyperparathyroidism and hypercalcemia among patients with a history of Li therapy or those undergoing Li treatment; (2) to determine whether Li treatment-associated factors (Li dose, duration of Li treatment, and serum Li concentration) are correlated with hyperparathyroidism and hypercalcemia; and (3) to establish cutoff values for predicting hyperparathyroidism and hypercalcemia.

## 2. Methods

### 2.1. Study Subjects

The participants in this study were consecutive individuals aged 18 years who underwent Li treatment. They were either inpatients or outpatients at the Department of Psychiatry or Department of Medicine at Tokyo Women's Medical University between April 2015 and March 2020. Patients with an estimated glomerular filtration rate (eGFR) below 45 mL/min/1.73 m^2^ and those concurrently experiencing disorders associated with Ca were excluded. In addition, individuals with unstable mental states that could hinder their understanding and completion of examinations were excluded. This retrospective cross-sectional study was conducted according to the research objectives and methodology outlined on the hospital website, allowing individuals to opt out at any time. This study adhered to the principles of the 1975 Declaration of Helsinki, as revised in 2013. Written informed consent was obtained from all participants, in compliance with the Ethics Committee of Tokyo Women's Medical University (approval number: 5683).

### 2.2. Background Factors

At the initiation of this study, we gathered information on the patients' age, sex, weight and height, diagnostic categories (BD, schizophrenia, depression, etc.), medical history (presence or absence of diabetes (DM) and hypertension (HTN)), Li dosage, duration of Li use, and hematological parameters. These parameters included serum levels of Li, intact parathyroid hormone (i-PTH), adjusted serum Ca (AdCa), inorganic phosphate (IP), magnesium (Mg), albumin (Alb), creatinine (Cre), thyroid-stimulating hormone (TSH), free thyroxine (fT4), and eGFR.

Blood samples were collected after at least 15 min of rest while the patients were seated. Ca, i-PTH, IP, Mg, Alb, Cre, TSH, and fT4 levels were measured using standard laboratory methods at our clinical laboratory center. Serum Li concentrations were measured using F28 tetraphenylporphyrin (Nipro Espa Li II, Osaka, Japan) at an external laboratory (SRL, Inc., Tokyo, Japan). AdCa was calculated using the following formula: AdCa = Ca − Alb + 4.0 (mg/dL) (if Alb was < 4.0 mg/dL). Estimated GFR was calculated using the following equation: eGFR (mL/min/1.73 m^2^) = 194 × creatinine − 1.094 × age − 0.287 (× 0.739 in case of females) [14].

### 2.3. Statistical Analysis

Demographic information of the patients, including age, sex, BMI, diagnostic categories, medical history, Li dosage, duration of Li use, and blood levels of Li, i-PTH, AdCa, IP, Mg, Alb, Cre, TSH, fT4, and eGFR, were analyzed. Group-wise mean comparisons were performed using a *t*-test or Wilcoxon rank-sum test. Normally distributed continuous variables were presented as the means ± standard deviation whereas non-normally distributed variables were expressed as medians with interquartile ranges (25th and 75th percentiles: IQR). Single regression analyses were conducted using Spearman's rank correlation to explore the correlation between i-PTH and AdCa levels and background factors. Subsequently, stepwise multiple regression analyses were performed, with the criteria for variable addition or removal set at a ρ value of 0.2. Receiver operating characteristic (ROC) curves were constructed to identify the cutoff values for the Li concentration in each group. Statistical significance was set at *p* < 0.05. The statistical analysis was carried out using JMP Pro 16.0.0 software (SAS Institute Inc., Cary, NC, USA).

## 3. Results

### 3.1. Study Subjects

**Figure 1:**
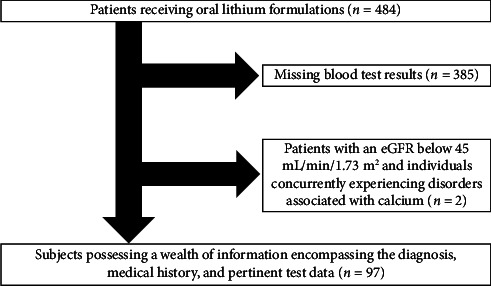
Study disposition.

Out of a pool of 484 individuals, 385 patients were excluded due to missing blood test results and 2 were excluded due to an eGFR below 45 mL/min/1.73 m^2^ (Figure 1). Consequently, the data from 97 patients were included in the analyses.

### 3.2. Background Factors

The clinical profiles and characteristics of the entire cohort of 97 patients are presented in Table 1. The mean age of the cohort was 57 ± 13 years, with a sex distribution of 41 males. Among the comorbidities, 11 patients presented with DM and 14 with HTN. Antihypertensive medications included Ca channel blockers in 11, angiotensin II receptor blockers in 3, furosemide in 1, and thiazide in 1. Moreover, 7 individuals were undergoing treatment for osteoporosis, 4 were receiving therapy for vitamin D deficiency, and 4 were undergoing treatment with bisphosphonates. None of the subjects were receiving estrogen therapy. The dose of Li ingestion was 600 (400–800) mg/day for 97 (60–158) months. The serum Li concentration was 0.54 ± 0.25 mg/dL, while the serum i-PTH and AdCa levels were 57 (48–75) pg/mL and 9.5 ± 0.4 mg/dL, respectively.

#### 3.2.1. Comparison of the Clinical Backgrounds Based on Serum Levels of i-PTH and AdCa

Among the 97 patients, 34 (35.1%) had serum i-PTH levels exceeding the upper limit of normal (65 pg/dL). The patients were stratified into two groups based on serum i-PTH levels: the hyper i-PTH group (serum i-PTH level ≥ 65 mg/dL) and the normal i-PTH group (serum i-PTH level < 65 mg/dL). The clinical profiles and characteristics of the two groups are shown in Table 2. Compared with the normal i-PTH group, the serum concentrations of Li (*p*=0.016) and Cre (*p*=0.036) in the hyper-PTH group were significantly elevated, while the eGFR showed a significant decrease (*p*=0.042).

Serum AdCa levels exceeded the upper limit of normal (10 mg/dL) in 9 patients (9.3%). The patients were stratified into two groups based on serum AdCa levels (hypercalcemia group, serum AdCa level ≥ 10 mg/dL; normocalcemia group, < 10 mg/dL). The clinical backgrounds and characteristics of the two groups are shown in Table 3. The serum Li concentration was significantly higher in the hypercalcemia group than that in the normocalcemia group (*p*=0.040).

#### 3.2.2. Single Correlation Analyses of Background Factors With Hyperparathyroidism and Hypercalcemia

Associations among background factors, hyperparathyroidism, and hypercalcemia were examined using Spearman's rank correlation coefficients. Both duration of Li administration (hyper i-PTH: ρ = 0.260,*p*=0.010 and hypercalcemia: ρ = 0.206,*p*=0.044) and serum Li concentration (hyper i-PTH: ρ = 0.274,*p*=0.006 and hypercalcemia: ρ = 0.232,*p*=0.022) showed significant and positive associations with hyper i-PTH and hypercalcemia (Table 4).

#### 3.2.3. Multiple Regression Analyses of Background Factors With Hyperparathyroidism and Hypercalcemia

Multiple regression analyses were performed using a stepwise method. Serum Li concentration, but not the duration of Li use, exhibited a statistically significant positive correlation with hyper PTH levels (*p*=0.044) and hypercalcemia (*p*=0.047); this correlation was independent of other factors (Table 5).

#### 3.2.4. Cutoff Values of Serum Li Concentration for Predicting Hyperparathyroidism and Hypercalcemia

**Figure 2:**
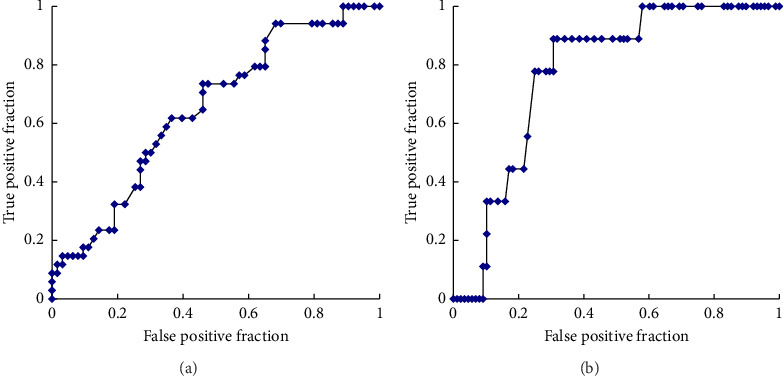
Receiver operating characteristic (ROC) curve for predicting hyperparathyroidism (a) and hypercalcemia (b).

ROC curve analyses were performed to determine the serum Li concentration cutoff value for predicting hyperparathyroidism and hypercalcemia. The analysis revealed a sensitivity of 74% and a specificity of 54% for predicting high serum i-PTH levels. In the ROC, the area under the curve (AUC) was 0.649, and the optimal cutoff value for a high serum i-PTH level was 0.52 mg/dL (Figure 2(a)). Furthermore, the analysis showed a sensitivity of 89% and specificity of 69% for predicting high serum AdCa levels, with an AUC of 0.773 and an optimal cutoff value of 0.62 mg/dL (Figure 2(b)).

## 4. Discussion

This study revealed four key findings. First, hyperparathyroidism and hypercalcemia were observed in 35.1% and 9.3% of the patients taking Li, respectively. Second, serum Li concentrations were significantly higher in the hyper i-PTH group than those in the normal i-PTH group and in the hypercalcemia group than those in the normocalcemia group. Third, the serum Li concentration exhibited significant positive correlations with hyperparathyroidism and hypercalcemia, independent of other factors. Fourth, the cutoff values of serum Li concentration for predicting hyperparathyroidism and hypercalcemia were 0.52 and 0.62 mEq/L, respectively. These data suggest the importance of monitoring the serum Li concentrations in patients administered this agent to prevent these conditions.

Our data revealed that the incidence of hyperparathyroidism and hypercalcemia in consecutive patients receiving Li treatment for psychiatric diseases was as high as 35.1% and 9.3%, respectively (Tables 2 and 3). Although we did not include a control group of patients with psychiatric disorders who were not taking Li, these incidences appeared to be elevated, confirming the possibility that Li may contribute to hyperparathyroidism and hypercalcemia. Although the mechanisms by which Li induces hyperparathyroidism and hypercalcemia are not fully understood, several proposed pathways may explain these effects. First, Li may stimulate the growth of parathyroid cells by activating the Wnt pathway, leading to the development of parathyroid hyperplasia and adenoma, primarily hyperplasia [15, 16]. Second, Li may interfere with Ca-sensing receptors (CaSRs) located on the surface of parathyroid cells [6, 17], which play a crucial role in regulating PTH secretion in response to changes in extracellular Ca levels. The disruption of CaSRs by Li can result in inappropriate PTH release. Third, Li may inhibit glycogen synthase kinase 3β, a negative regulator of the Wnt pathway, promoting the growth of parathyroid cells [18]. Hypercalcemia can be induced by hyperparathyroidism through increased Ca release from the bone and enhanced renal reabsorption. In addition, Li may cause nephrogenic diabetes insipidus by inhibiting the expression of aquaporin channels, mainly aquaporin 2, in the renal collecting tubules, potentially leading to hypercalcemia through dehydration [11].

Comparing our findings with those of previous studies, the incidence of hypercalcemia in our study (9.3%) was relatively low. This phenomenon is speculated to have resulted from the omission of ionized Ca measurements in our study. Previous investigations have reported elevated ionized Ca levels (25%–42.3%) in patients exposed to Li [5, 9, 10]. We hypothesized that if ionized Ca was measured in our study, similar or potentially higher results might have been obtained. The prevalence of patients with uncompromised renal function in our study who exhibited increased urinary Ca excretion might have contributed to the maintenance of Ca levels within the normal range in the bloodstream.

Once hypercalcemia and hyperparathyroidism are diagnosed, the clinician must decide on either cessation of Li and consideration of alternative psychiatric medication(s); surgical excision of abnormal parathyroid tissue, mainly in typical hyperparathyroidism phenotypes with a unique adenoma; or treatment with calcimimetic drugs such as cinacalcet [19].

Before discussing the limitations of our study, it is important to clarify aspects of data availability and clinical monitoring practices. In this retrospective cohort, serum Ca was routinely measured in all patients receiving Li therapy as part of standard clinical practice in Japan. However, i-PTH testing was not performed in all cases, and the missing data in this study primarily refer to the absence of i-PTH measurements. It is also worth noting that monitoring practices for Li vary internationally; for example, routine i-PTH testing is not consistently performed in all countries, particularly in asymptomatic patients. This variation should be taken into account when interpreting cross-study comparisons.

It is well established that blood Li concentrations have a narrow optimal range. Levels below this range may not yield the desired therapeutic effects whereas those exceeding this range may lead to Li toxicity. Therefore, a narrow therapeutic range between 0.6 and 1.2 mEq/L has been recommended [19]. Notably, in this study, when the Li concentration exceeded 0.52 mEq/L, there was a significant association with hyperparathyroidism, and when it exceeded 0.62 mEq/L, there was a significant correlation with hypercalcemia. The occurrence of these conditions is deemed possible even within the conventional optimal therapeutic range of Li (0.52–1.2 mEq/L), emphasizing the need for caution in its use. However, adhering to the Li concentration to less than 0.52 mEq/L may increase the likelihood of deteriorating mental states. In the cases we encountered, the cessation of Li led to a decrease in i-PTH and Ca levels. However, deterioration in the mental state was also observed, necessitating the resumption of Li administration (data not shown). For patients undergoing Li therapy, careful monitoring of both serum Ca and Li levels is essential. Multidisciplinary collaboration, including timely consultation with psychiatrists and endocrine surgeons, is recommended to ensure optimal clinical management.

The present study has several limitations. First, the sample size was relatively small and this was a single-center study with subjects from a single ethnic population (Japanese). Second, the presence of patients with secondary hyperparathyroidism due to chronic kidney disease may have influenced our data as renal function was slightly but significantly worse in the hyper i-PTH group than that in the normal i-PTH group (Table 2). However, the association between serum Li concentration and hypercalcemia cannot be dismissed because these associations are independent of renal function (Table 5). Third, the lack of assessment of parathyroid gland morphology precludes the establishment of a differential diagnosis for hyperparathyroidism, including primary hyperparathyroidism. Fourth, there was no control group of patients with psychiatric disorders who did not receive Li therapy. Therefore, a definitive assertion that the observed increase in the prevalence of hyperparathyroidism and hypercalcemia in this study can be attributed to Li use, not the psychiatric disorders themselves, cannot be made, although such findings have not been previously reported. Fifth, as this study was a retrospective cross-sectional investigation, it precluded the assessment of both pretreatment status and the changes over time in serum Ca, i-PTH, and Li concentrations. Larger-scale prospective studies are needed to investigate the significance of assessing blood levels of Li, Ca, and i-PTH. Similarly, the inability to assess vitamin D deficiency is considered a significant limitation. Age-related hyperparathyroidism must also be considered as a confounding factor to be excluded.

In conclusion, the present study confirmed a high incidence of hyperparathyroidism and hypercalcemia in patients taking Li. The results also demonstrated that serum Li concentration was significantly and positively correlated with hyperparathyroidism and hypercalcemia, independent of other factors. The cutoff values of serum Li concentration for predicting hyperparathyroidism and hypercalcemia were 0.52 and 0.62 mEq/L, respectively. Clinicians should be aware that Li treatment can cause hyperparathyroidism and hypercalcemia. In addition, it may be recommended to follow not only serum Ca concentrations but also serum Li concentrations in patients undergoing Li treatment. Serum Li concentrations higher than 0.52 mEq/L may be associated with hyperparathyroidism.

## Figures and Tables

**Table 1:** Characteristics of the study subjects.

*N*	97
Age (y.o.)	57 ± 13
Gender (male/female)	41/56
BMI (kg/m^2^)	24.1 ± 4.6
Bipolar	72
Schizophrenia	14
Depression	10
Other	1
History of DM	11
History of HTN	14
History of osteoporosis	7
Li dose (mg/day)	600 (400–800)
Duration of Li medication (month)	97 (60–158)
Li (mEq/L)	0.54 ± 0.25
i-PTH (pg/mL)	57 (48–75)
Adjusted Ca (mg/dL)	9.5 ± 0.4
P (mg/dL)	3.2 (2.9–3.7)
Mg (mg/dL)	1.8 (1.7–1.9)
Alb (g/dL)	4.4 (4.2–4.7)
Cre (mg/mL)	0.81 ± 0.18
eGFR (mL/min/1.73 m^2^)	63.5 (53.6–73.8)
TSH (μIU/mL)	2.31 (1.30–3.71)
fT4 (ng/dL)	1.10 (0.98–1.21)

*Note:* HTN, hypertension; Li, lithium; Cre, creatinine; i-PTH, intact parathyroid hormone; fT4, free thyroxin.

Abbreviations: BMI, body mass index; DM, diabetes mellitus; eGFR, estimated glomerular filtration rate; TSH, thyroid stimulating hormone.

**Table 2:** Comparisons of the clinical backgrounds between the hyper and normal i-PTH groups.

	Hyper i-PTH (≥ 65 mg/dL)	Normal i-PTH (< 65 mg/dL)	*p* value
*N*	34	63	—
Age (y.o.)	60 ± 14	55 ± 14	0.059
Gender (male/female)	15 (44%)	26 (41%)	0.567
BMI (kg/m^2^)	24.1 ± 4.7	24.0 ± 4.6	0.927
Bipolar	29	43	0.966
Schizophrenia	3	11	—
Depression	2	8	—
Other	0	1	—
History of DM	6	5	—
History of HTN	2	6	—
Li dose (mg/day)	600 (400–800)	600 (400–600)	0.731
Duration of Li medication (month)	114 (92–187)	85 (44–131)	0.246
Li (mEq/L)	0.61 (0.45–0.71)	0.50 ± 0.23	0.016
i-PTH (pg/mL)	80 (72–93)	50 ± 9	< 0.001
Adjusted Ca (mg/dL)	9.5 ± 0.4	9.5 ± 0.4	0.882
IP (mg/dL)	3.2 (3.0–3.4)	3.2 ± 0.6	0.857
Mg (mg/dL)	1.8 (1.7–1.9)	1.8 (1.7–1.9)	0.280
Alb (g/dL)	4.4 ± 0.3	4.3 (4.2–4.7)	0.583
Cre (mg/mL)	0.83 (0.70–1.04)	0.79 ± 0.17	0.036
eGFR (mL/min/1.73 m^2^)	57.4 (51.3–71.3)	66.9 (57.1–75.7)	0.042
TSH (μIU/mL)	2.60 (1.56–3.24)	2.11 (1.23–3.74)	0.326
fT4 (ng/dL)	1.05 (0.99–1.19)	1.13 (0.97–1.24)	0.343

*Note:* HTN, hypertension; Li, lithium; i-PTH, intact parathyroid hormone; Ca, calcium; Mg, magnesium; Alb, albumin; Cre, creatinine; fT4, free thyroxine.

Abbreviations: BMI, body mass index; DM, diabetes mellitus; eGFR, estimated glomerular filtration rate; IP, inorganic phosphate; TSH, thyroid-stimulating hormone.

**Table 3:** Comparisons of the clinical backgrounds between the hypercalcemia and normocalcemia groups.

	Hypercalcemia (≥ 10 mg/dL)	Normocalcemia (< 10 mg/dL)	*p* value
*N*	9	88	—
Age (y.o.)	64 ± 12	56 ± 14	0.102
Gender (male/female)	2/7	39/49	0.284
BMI (kg/m^2^)	25.9 ± 5.3	23.8 ± 4.5	0.221
Bipolar	7	65	0.638
Schizophrenia	1	13	—
Depression	1	9	—
Other	0	1	—
History of DM	3	8	—
History of HTN	1	7	—
Li dose (mg/day)	655 ± 250	600 (400–800)	0.187
Duration of Li medication (month)	98 (90–156)	96 (52–158)	0.497
Li (mEq/L)	0.71 ± 0.13	0.53 (0.37–0.67)	0.040
i-PTH (pg/mL)	64 ± 24	57 (48–76)	0.899
Adjusted Ca (mg/dL)	10.1 (10.1–10.2)	9.4 (9.2–9.7)	< 0.001
IP (mg/dL)	3.3 ± 0.3	3.2 (2.9–3.6)	0.829
Mg (mg/dL)	1.8 ± 0.2	1.8 (1.7–1.9)	0.135
Alb (g/dL)	4.5 ± 0.2	4.4 (4.2–4.6)	0.289
Cre (mg/mL)	0.85 ± 0.13	0.82 ± 0.18	0.604
eGFR (mL/min/1.73 m^2^)	55.7 (44.9–67.5)	65.2 (55.8–74.7)	0.180
TSH (μIU/mL)	3.27 ± 1.57	2.14 (1.30–3.59)	0.714
fT4 (ng/dL)	1.08 ± 0.16	1.12 (0.99–1.22)	0.681

*Note:* HTN, hypertension; Li, lithium; i-PTH, intact parathyroid hormone; Ca, calcium; Mg, magnesium; Alb, albumin; Cre, creatinine; fT4, free thyroxine.

Abbreviations: BMI, body mass index; DM, diabetes mellitus; eGFR, estimated glomerular filtration rate; IP, inorganic phosphate; TSH, thyroid-stimulating hormone.

**Table 4:** Single correlation analyses of background factors with hyper i-PTH and hypercalcemia.

	Hyper i-PTH		Hypercalcemia	
	ρ	*p*	ρ	*p*
Li dose	0.142	0.162	0.033	0.747
Duration of Li medication	0.260	0.010	0.206	0.044
Li	0.274	0.006	0.232	0.022
Mg	0.167	0.109	0.052	0.619
Cre	0.050	0.652	0.056	0.580
fT4	0.013	0.894	−0.004	0.967

*Note:* i-PTH, intact parathyroid hormone; Li, Lithium; Mg. magnesium; fT4, free thyroxine.

Abbreviation: eGFR, estimated glomerular filtration rate.

**Table 5:** Multiple regression analyses of background factors with hyper i-PTH and hypercalcemia.

	Hyper i-PTH		Hypercalcemia	
	β	*p*	β	*p*
Li dose	−0.002	0.982	—	—
Duration of Li medication	0.037	0.730	0.001	0.125
Li	0.257	0.044	0.001	0.047
i-PTH	—	—	2.379	0.096
Adjusted Ca	−0.127	0.240	—	—
Mg	0.089	0.424	—	—

*Note:* Hyper i-PTH: *R*^2^ = 0.090, *p*=0.134 for the entire model. Hypercalcemia: *R*^2^ = 0.093, *p*=0.030 for the entire model. i-PTH, intact parathyroid hormone; Li, lithium; Ca, calcium; Cre, creatinine.

## Data Availability

Restrictions apply to the availability of some or all data generated or analyzed during this study to preserve patient confidentiality or because they were used under license. The corresponding author will request details regarding the restrictions and conditions under which access to the data may be provided.
